# Beyond Seizure Suppression: Caffeic Acid Phenethyl Ester and 10-Hydroxy-2-Decenoic Acid Improve Anxiety-like and Cognitive Behaviors in PTZ-Challenged Zebrafish

**DOI:** 10.3390/vetsci13101050

**Published:** 2026-10-07

**Authors:** Muhammet Enis Yonar, Mücahit Eroğlu, Ünal İspir, Sibel Silici, Seda Özcan, Feyza Karahüseyinoğlu, Tuba Çakmak Duran, Cemal Orhan, Serpil Mişe Yonar

**Affiliations:** 1 Department of Aquaculture, Faculty of Fisheries, Fırat University, Elazig 23200, Türkiye; 241128102@firat.edu.tr (F.K.); tduran@istac.istanbul (T.Ç.D.); s_mise@firat.edu.tr (S.M.Y.); 2 Department of Basic Sciences, Faculty of Fisheries, Fırat University, Elazig 23200, Türkiye; meroglu44@firat.edu.tr; 3 Department of Aquaculture, Faculty of Agriculture, Malatya Turgut Özal University, Malatya 44210, Türkiye; unal.ispir@ozal.edu.tr; 4 Department of Agricultural Biotechnology, Faculty of Agriculture, Erciyes University, Kayseri 38039, Türkiye; silicis@erciyes.edu.tr; 5 Nutral Therapy Co., Erciyes Technopark, Kayseri 38039, Türkiye; 6 Department of Child and Adolescent Mental Health and Diseases, Children’s Hospital, Faculty of Medicine, Ege University, Izmir 35100, Türkiye; seda.sari@ege.edu.tr; 7 Department of Animal Nutrition, Faculty of Veterinary Medicine, Fırat University, Elazig 23200, Türkiye; corhan@firat.edu.tr

**Keywords:** caffeic acid phenethyl ester, 10-hydroxy-2-decenoic acid, neurobehavioral dysfunction, pentylenetetrazol, seizure, zebrafish

## Abstract

Epilepsy is associated with recurrent seizures and behavioral and cognitive disturbances, highlighting the importance of assessing behavioral outcomes alongside seizure severity. Caffeic acid phenethyl ester (CAPE) has shown neuroprotective activity, whereas evidence for the neurobehavioral effects of 10-hydroxy-2-decenoic acid (10-HDA) remains limited. This study investigated their individual and combined dietary effects on seizure-like behavior and behavioral test performance in adult zebrafish (*Danio rerio*) challenged with pentylenetetrazol (PTZ). Fish received experimental diets for 8 weeks before exposure to 15 mM PTZ. A dietary valproic acid group was included as a comparator. Outcomes included behavioral seizure scores, the area under the seizure score–time curve (AUC), and performance in the novel tank diving and Y-maze tests. Compared with PTZ alone, CAPE, 10-HDA, and their combination reduced seizure scores and AUC values and increased upper-zone and novel-arm exploration (*p* < 0.05). The combined group spent more time in the novel arm than either single-compound group (*p* < 0.05), although its advantages varied across endpoints and pharmacological synergy was not established. Overall, dietary CAPE and 10-HDA were associated with lower behavioral seizure scores and improved test performance following acute PTZ challenge. Further studies are needed to distinguish these responses from independent effects on baseline behavior and the influence of seizure severity and motor function.

## Simple Summary

Epilepsy can affect behavior, learning, and memory as well as cause seizures. This study explored two compounds found in bee products, caffeic acid phenethyl ester (CAPE) and 10-hydroxy-2-decenoic acid (10-HDA), using adult zebrafish. Fish received one or both compounds in their diets for eight weeks before exposure to a chemical that induces seizure-like behavior. Compared with fish exposed to this chemical without supplementation, supplemented fish had lower seizure scores, explored the upper part of a new tank more, and spent more time in a previously inaccessible maze arm. The combination performed better than either compound alone on some measures, but not consistently enough to suggest a general advantage or establish synergy. These findings encourage further research into both compounds. However, greater exploration does not necessarily mean reduced anxiety or better memory. Testing took place shortly after the seizure challenge, and the compounds’ effects on behavior without that challenge were not assessed. Further work is therefore needed to determine how much the observed changes reflect reduced seizure severity, changes in swimming ability, or other behavioral effects.

## 1. Introduction

Epilepsy is one of the most prevalent chronic neurological disorders worldwide and continues to impose a substantial health burden across different age groups and geographical regions. Epidemiological studies show considerable variation in prevalence and incidence across populations, reflecting differences in demographic, socioeconomic, and healthcare-related factors [1,2,3,4]. At the global level, the burden of epilepsy remains considerable, with its consequences extending beyond recurrent seizures to long-term healthcare needs and disease-related disability [5]. Despite substantial advances in antiseizure therapies, effective seizure control cannot be achieved in a considerable proportion of patients, making drug-resistant epilepsy a persistent challenge in clinical management [6,7,8]. Pharmacoresistance is a complex, multifactorial phenomenon involving multiple biological mechanisms that can limit the effectiveness of conventional antiseizure medications [9,10]. The continuing burden of refractory epilepsy has therefore intensified the search for alternative therapeutic strategies and novel bioactive compounds with anticonvulsant potential [11,12,13].

Importantly, the therapeutic challenge of epilepsy extends beyond seizure control. Epilepsy is increasingly recognized as a disorder associated with a broad spectrum of neurobehavioral and cognitive comorbidities that can substantially affect daily functioning and quality of life [14,15,16]. Anxiety and other behavioral disturbances frequently accompany epilepsy, while deficits in learning, memory, attention, and other cognitive domains constitute important components of its clinical phenotype [17,18,19]. Evidence from experimental models further supports a close association between seizure susceptibility and alterations in anxiety- and stress-related behaviors, learning, and memory, suggesting that these manifestations form part of the broader neurobehavioral phenotype of epilepsy rather than simply representing secondary consequences of seizure episodes [15,20]. Notably, successful seizure control does not necessarily resolve these comorbidities, and their management remains challenging. Moreover, antiseizure medications themselves can adversely affect cognition, mood, and behavior, although the nature and magnitude of these effects vary among drugs and individuals [21,22,23,24]. Such cognitive and behavioral adverse effects have also been reported for newer antiseizure medications, indicating that seizure suppression and preservation of neurobehavioral function do not always occur in parallel [25,26]. Accordingly, assessing emerging therapeutic candidates should extend beyond anticonvulsant efficacy to include their capacity to preserve or improve behavioral and cognitive function.

Zebrafish (*Danio rerio*) has emerged as a valuable vertebrate model for epilepsy research and neuropharmacological screening because it enables reproducible characterization of seizure-related phenotypes together with a broad range of behavioral outcomes [27,28]. Among chemically induced seizure models, pentylenetetrazol (PTZ), a GABA_A receptor antagonist, is widely used to evoke robust seizure-like activity in zebrafish, producing concentration-dependent alterations in locomotor behavior and neuronal activity [29,30]. Importantly, detailed characterization in adult zebrafish has demonstrated that PTZ exposure induces a progressive, readily quantifiable behavioral seizure phenotype, supporting its utility for assessing the anticonvulsant potential of candidate compounds [31]. Beyond seizure scoring, adult zebrafish allow examination of seizure-associated neurobehavioral disturbances within the same experimental framework. The novel tank diving test is particularly useful for characterizing anxiety-like responses through behavioral measures such as vertical exploration, bottom dwelling, latency, freezing, and darting, although careful consideration of individual behavioral variability and test standardization is essential for robust interpretation [32,33,34,35]. Maze-based paradigms can also investigate the cognitive consequences of seizure activity, with zebrafish models demonstrating their utility for evaluating epilepsy-associated learning and memory dysfunction [36]. Collectively, the integration of seizure scoring with complementary behavioral assays provides a multidimensional approach that extends phenotypic assessment beyond seizure intensity alone and enables simultaneous evaluation of anxiety-like and cognitive alterations. This combination of experimentally tractable seizure induction and quantifiable neurobehavioral endpoints makes adult zebrafish a particularly useful platform for screening compounds with potential effects on both seizure activity and its associated behavioral consequences [27,28].

Caffeic acid phenethyl ester (CAPE), a bioactive phenolic compound, has attracted considerable attention for its neuroprotective potential across a variety of neurological and neurotoxic conditions [37,38,39]. Of particular relevance to epilepsy, CAPE has shown protective effects against chemically induced seizures. In a PTZ-induced seizure model in mice, CAPE attenuated seizure-associated alterations and protected the central nervous system, providing early evidence of its anticonvulsant and neuroprotective potential [40]. CAPE also reduced apoptotic neuronal damage following PTZ-induced status epilepticus in the developing rat brain [41], while more recent evidence demonstrated protection against nicotine-induced seizures in mice [42]. Its neuroprotective activity has additionally been demonstrated across experimental models of neurological injury and neurodegeneration [43,44,45,46,47]. Importantly, CAPE has been reported to ameliorate cognitive deficits under different experimental conditions, further supporting its potential relevance to seizure-associated behavioral dysfunction [48,49,50].

10-Hydroxy-2-decenoic acid (10-HDA), a characteristic bioactive fatty acid of royal jelly, has recently attracted interest for its potential effects on the central nervous system. However, evidence regarding its neurobehavioral and anticonvulsant properties remains limited compared with CAPE. Recent findings demonstrated that 10-HDA exerted neuroprotective effects in an experimental model of traumatic brain injury by attenuating neuronal damage [51]. Nevertheless, its effects on seizure susceptibility and seizure-associated behavioral disturbances, including anxiety-like behavior and cognitive dysfunction, remain largely unexplored, particularly in zebrafish models.

Although CAPE has shown neuroprotective and seizure-related effects in experimental models, evidence for the neurobehavioral effects of 10-HDA remains limited. The effects of dietary CAPE and 10-HDA, individually and in combination, on seizure-like behavior and subsequent behavioral test performance in adult zebrafish remain poorly characterized. This study addresses this gap by examining both compounds alone and together within the same dietary protocol, combining behavioral seizure assessment with novel tank and Y-maze testing. We hypothesized that supplemented fish would show lower behavioral seizure scores and improved performance in these tests compared with fish exposed to PTZ without supplementation. By integrating these complementary endpoints, this study aimed to assess seizure-like behavior and novel tank and Y-maze performance in supplemented, PTZ-challenged fish relative to fish exposed to PTZ without supplementation.

## 2. Materials and Methods

### 2.1. Animals and Ethical Approval

A total of 810 adult zebrafish (*Danio rerio*), aged 7–8 months, were obtained from a commercial supplier and acclimated to the laboratory conditions before initiation of the experiment. Fish were randomly allocated to experimental groups, with 30 individuals maintained in each aquarium per replicate. The entire experimental protocol followed institutional guidelines for the care and use of experimental animals and was approved by the Fırat University Local Ethics Committee for Animal Experiments (Approval No. 2024/10-01).

### 2.2. Chemicals and Preparation of Experimental Diets

Caffeic acid phenethyl ester (CAPE), 10-hydroxy-2-decenoic acid (10-HDA), valproic acid (VPA), pentylenetetrazol (PTZ), and the remaining chemicals used throughout the experiment were obtained from commercial sources (Sigma-Aldrich, St. Louis, MO, USA).

A commercial particulate aquarium feed was used as the basal diet. For the single-compound diets, CAPE or 10-HDA was dissolved in 1 mL of 30% ethanol prepared with distilled water and incorporated into 1 kg of feed at a nominal inclusion level of 10 mg/kg. For the combined diet, 10 mg CAPE and 10 mg 10-HDA were dissolved together in the same 1 mL of 30% ethanol and incorporated into 1 kg of feed, providing 10 mg/kg of each compound. For VPA, the amount of the commercial preparation required was calculated based on its active ingredient content. A nominal 100 µM aqueous VPA preparation was made using distilled water, and the entire 1 L was incorporated into 1 kg of feed, corresponding to a nominal dietary inclusion level of 100 µmol/kg feed. The preparations were applied gradually to the respective diets with continuous mixing to promote uniform distribution. These inclusion levels represent the amounts added during preparation rather than analytically verified concentrations in the finished diets.

Following supplementation, the diets were spread in a thin layer on trays and air-dried at room temperature for 48 h to allow solvent evaporation. The feed was mixed periodically during drying to ensure uniform exposure to air. Experimental diets were prepared weekly and stored at 4 °C in dark glass containers until use. Fish in the control and PTZ groups received the same basal feed without supplementation.

The selection of CAPE and 10-HDA inclusion levels was informed by previous dietary studies involving royal jelly in zebrafish [52] and caffeic acid in Nile tilapia [53]. These studies provided an indirect rationale for dose selection because they examined different preparations or compounds and, in the latter case, a different fish species. Accordingly, the selected inclusion levels were experimental doses rather than established effective doses for CAPE and 10-HDA in the adult zebrafish PTZ model. An 8-week feeding period was used to evaluate the effects of sustained dietary supplementation before the acute PTZ challenge. PTZ exposure conditions were adapted from Mussulini et al. [31]. Feas et al. [54] informed the selection of VPA as a reference antiseizure agent, but their larval exposure protocol did not directly validate the dietary dose or administration period used here.

The proximate composition of the experimental diets was determined using standard analytical procedures. Moisture, crude protein, crude lipid, crude fiber, crude ash, and nitrogen-free extract were evaluated according to AOAC procedures. Crude protein was determined using the micro-Kjeldahl method, crude lipid by solvent extraction, crude fiber using a cellulose determination system, and ash by incineration in a muffle furnace. Digestible energy values were estimated using established energy coefficients for fish-feed ingredients.

### 2.3. Experimental Design and PTZ Exposure

The experiment comprised nine groups and was conducted in three separate experimental runs at different times. In each run, fish were randomly allocated to the nine groups, with 30 fish per group. This yielded 90 fish per group across the three runs and 810 fish in the overall experiment.

The experimental groups were organized as follows:

Control (C): Fish received the basal diet for 8 weeks.
Positive control (PC): Fish received a diet supplemented with VPA at a nominal inclusion level of 100 µmol/kg feed for 8 weeks and were subsequently exposed to 15 mM PTZ.
CAPE: Fish received a diet containing CAPE at 10 mg/kg feed for 8 weeks.
10-HDA: Fish received a diet containing 10-HDA at 10 mg/kg feed for 8 weeks.
CAPE+10-HDA: Fish received a diet containing both CAPE and 10-HDA at 10 mg/kg feed each for 8 weeks.
PTZ: Fish received the basal diet for 8 weeks and were subsequently exposed to 15 mM PTZ.
CAPE+PTZ: Fish received CAPE at 10 mg/kg feed for 8 weeks and were subsequently exposed to 15 mM PTZ.
10-HDA+PTZ: Fish received 10-HDA at 10 mg/kg feed for 8 weeks and were subsequently exposed to 15 mM PTZ.
CAPE+10-HDA+PTZ: Fish received CAPE and 10-HDA at 10 mg/kg feed each for 8 weeks and were subsequently exposed to 15 mM PTZ.

Although the overall experimental design included non-PTZ groups receiving CAPE, 10-HDA, or their combination, these groups formed part of the broader experimental protocol for evaluating the effects of dietary supplementation under basal conditions. The behavioral component of the present study focused specifically on PTZ-induced seizure activity and associated behavioral alterations, and on the effects of dietary CAPE and 10-HDA on these outcomes in PTZ-challenged fish. Accordingly, behavioral assessments were restricted to the C, PC, PTZ, CAPE+PTZ, 10-HDA+PTZ, and CAPE+10-HDA+PTZ groups. Behavioral measurements were not collected from the non-PTZ CAPE, 10-HDA, and CAPE+10-HDA groups, which were included for nonbehavioral assessments within the broader project. All 90 fish in each of the six groups selected for behavioral testing were assessed individually; no subset was selected.

Throughout the 8-week feeding period, fish were offered their respective experimental diets four times daily at 09:00, 12:00, 15:00, and 18:00, corresponding to approximately 5% of body weight per day. To maintain water quality, we siphoned approximately 10% of the aquarium water daily to remove uneaten feed and metabolic waste and replaced it with fresh water at the same temperature. Mean water temperature, pH, and dissolved oxygen were maintained at 25 ± 1 °C, 7.2 ± 0.3, and 6.8 ± 0.2 mg/L, respectively.

One day after completion of the 8-week feeding period, fish assigned to PTZ-challenged groups were immersed in 15 mM PTZ for 20 min. Behavioral responses were video-recorded for a total of 60 min from the onset of PTZ exposure. The initial 0–20 min interval was used for quantitative seizure assessment, while monitoring continued for the remaining 40 min to document late seizure-like responses and assess fish welfare. Immediately after this observation period, fish underwent the novel tank diving test, followed by the Y-maze test, with no additional waiting period between the behavioral tests.

### 2.4. Behavioral Assessment

#### 2.4.1. Assessment of PTZ-Induced Seizure Activity

Following the 8-week feeding period, behavioral assessments were performed in zebrafish exposed to 15 mM PTZ. PTZ-induced seizure activity was evaluated using established behavioral seizure scales for zebrafish [31,55,56]. Seizure-like responses during PTZ exposure were classified using a phenotypic scoring system based on the progressive severity of motor manifestations.

Seizure severity was scored according to the following scale: (0) brief swimming movements; (1) increased swimming activity accompanied by an increased frequency of opercular movements; (2) rapid swimming with irregular side-to-side movements; (3) circular swimming movements; (4) clonic seizure-like behavior characterized by loss of posture, including lateral or dorsal turning, accompanied by abnormal rhythmic contractions of the entire body musculature; (5) tonic seizure-like behavior characterized by immobility at the bottom of the tank and body rigidity; and (6) death. Supplementary Video S1 shows representative PTZ-induced seizure behaviors corresponding to the scoring criteria.

Seizure scores were recorded at 0, 60, 120, 180, 240, 300, 600, and 1200 s after the onset of PTZ exposure. The scoring system was used to quantify the progressive severity of PTZ-induced motor manifestations, ranging from mild alterations in swimming activity to clonic and tonic seizure-like behaviors and death. To integrate seizure severity and its temporal persistence into a single quantitative measure, the area under the seizure score–time curve (AUC) was calculated automatically in GraphPad Prism 11.0 over the 0–1200 s observation period. AUC represents the area under the individual seizure score-versus-time curve; therefore, higher AUC values indicate a greater cumulative seizure burden over the observation period, whereas lower values indicate a lower overall seizure burden. AUC values were expressed as scores. Higher AUC values indicated a greater cumulative seizure burden during the observation period.

#### 2.4.2. Novel Tank Diving Test

The novel tank diving test began immediately after the 60-min observation period following PTZ exposure. Individual zebrafish were transferred to a 1.5-L novel tank positioned on a stable, level surface. For behavioral observation, the water column was visually divided into three vertical zones: bottom, middle, and top.

Each fish was observed for 6 min after introduction to the novel environment, and two observers independently evaluated the behavioral recordings. The assessed parameters included the number of entries into the upper zone, time spent in each vertical zone, latency to first entry into the upper zone, darting, and freezing behavior. These endpoints were used collectively to characterize exploratory activity and anxiety-like responses. Supplementary Video S2 provides representative recordings of the experimental setup and behavioral assessment. Data obtained from the recordings were subsequently used for statistical comparisons among the experimental groups.

#### 2.4.3. Y-Maze Test

The Y-maze procedure was conducted immediately after the 6-min novel tank diving test, with no additional waiting period between tests. Novel-arm exploration was assessed using a glass Y-maze consisting of three arms, each measuring 25 × 8 × 15 cm. To minimize interference from uncontrolled external visual stimuli, the outer walls of the maze were covered with black adhesive film. The maze was filled with 3 L of water obtained from the corresponding holding aquaria. Distinct geometric visual cues consisting of light-colored circles, triangles, and quadrilaterals against a black background were positioned along the vertical walls of the maze arms, with a different shape assigned to each arm. The cue arrangement remained unchanged between the two trials and across fish.

The procedure used a two-trial Y-maze design to assess exploration of a previously inaccessible arm. Dellu et al. [57] provided the conceptual background for this approach in rodents, while Cognato et al. [56] investigated a two-trial Y-maze memory task specifically in zebrafish. The apparatus and procedures used in the present study are described below. The three arms were designated as the start arm, novel arm, and other arm. Each fish was initially introduced into the start arm, which remained accessible throughout the test. During trial 1, the novel arm was closed, whereas the start and other arms remained accessible. During trial 2, the novel arm was opened, allowing access to all three arms. The central region of the maze was considered a neutral zone and was excluded from arm-specific analyses.

Individual fish were allowed to explore the maze for 5 min. The behavioral variables recorded included time spent in the start, other, and novel arms; number of entries into the familiar and novel arms; and latency to first entry into the novel arm. These parameters were used to characterize exploration of the previously inaccessible arm and overall maze activity. Representative recordings of the experimental procedure are provided in Supplementary Video S3.

### 2.5. Statistical Analysis

Statistical analyses were performed using IBM SPSS Statistics version 25.0 and GraphPad Prism version 11.0. Data are presented as mean ± standard error of the mean (SEM) or median (minimum–maximum), as appropriate. Normality of data distribution was assessed using the Shapiro–Wilk test, while homogeneity of variances was evaluated using Levene’s and Bartlett’s tests. For normally distributed data with homogeneous variances, differences among experimental groups were analyzed using one-way analysis of variance (ANOVA), followed by Tukey’s honestly significant difference (HSD) test for multiple comparisons. When the assumption of homogeneity of variance was violated, Welch’s ANOVA was applied, followed by Tamhane’s T2 test for pairwise comparisons. Non-normally distributed data were analyzed using the Kruskal–Wallis test followed, where appropriate, by Dunn’s multiple-comparison test. AUC values were statistically compared among experimental groups using the appropriate parametric or non-parametric procedures described above. Statistical significance was accepted at *p* < 0.05.

For behavioral analyses, the individual fish was considered the observational unit (*n* = 90 per group); experimental run was not included as a random factor because all three runs were conducted using the same standardized environmental, husbandry, and behavioral testing conditions.

## 3. Results

### 3.1. Chemical Composition of the Diets

Table 1 presents the proximate composition and digestible energy content of the experimental diets. The diets showed comparable levels of crude protein, crude lipid, crude ash, and moisture, with a consistent digestible energy content of 3500 kcal/kg. Overall, the experimental diets were nutritionally comparable, indicating that the observed biological differences were primarily associated with the experimental treatments rather than differences in the proximate composition or energy content of the diets.

### 3.2. Behavioral Assessments

#### 3.2.1. The Tonic–Clonic Seizure

**Figure 1:**
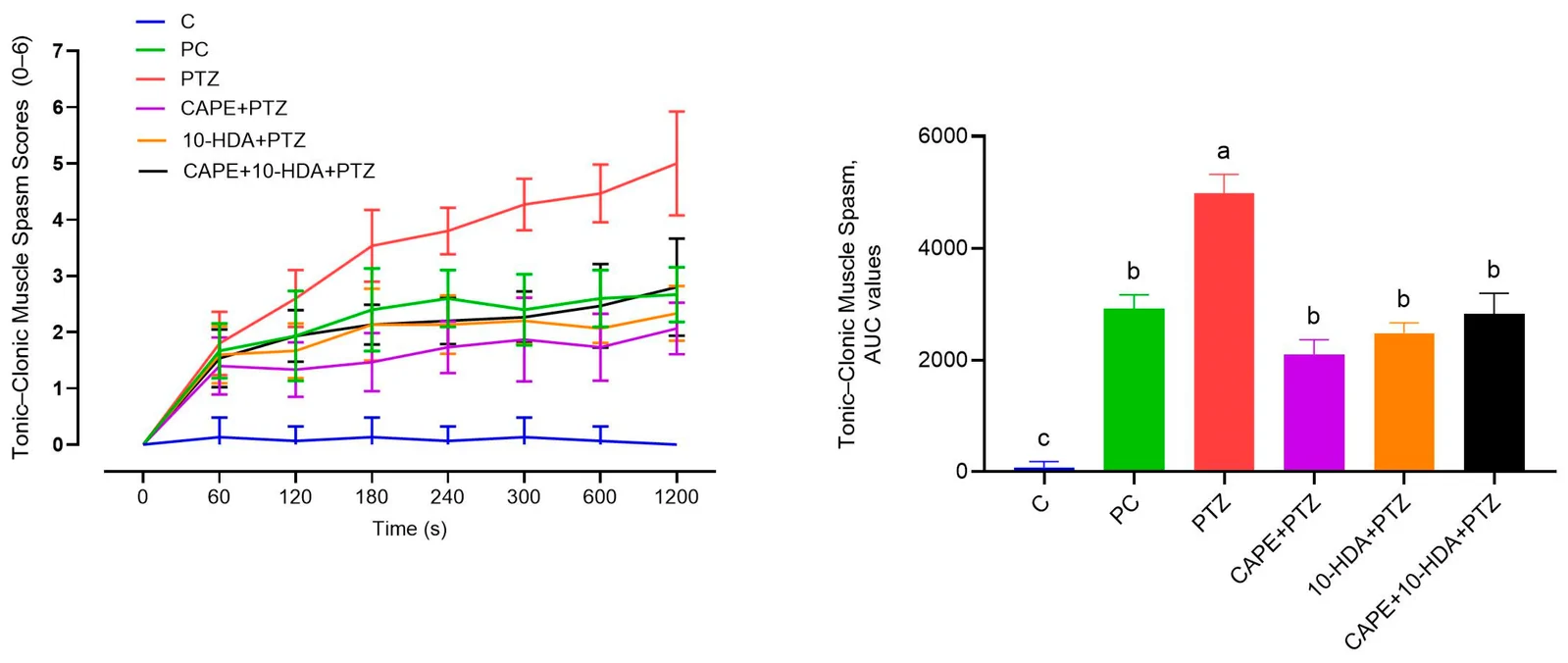
Behavioral seizure scores and area under the seizure score–time curve (AUC) in the control and PTZ-challenged groups. The left panel shows seizure scores recorded over 0–1200 s, and the right panel shows AUC values calculated over the same interval. Seizure severity was assessed using the following scale: (0) brief swimming movements; (1) increased swimming activity and opercular movements; (2) rapid swimming with irregular side-to-side movements; (3) circular swimming; (4) clonic seizure-like behavior with loss of posture and rhythmic whole-body contractions; (5) tonic seizure-like behavior with immobility at the bottom and body rigidity; and (6) death. Seizure scores are dimensionless, time is expressed in seconds, and AUC values are expressed as scores. Data are presented as mean ± SEM. All 90 fish per group were assessed (30 fish in each of three experimental replicates). In the AUC panel, groups with the same lowercase letter do not differ significantly, whereas groups with different letters differ significantly (*p* < 0.05).

Figure 1 shows PTZ-induced tonic–clonic seizure scores in the experimental groups, along with area under the curve (AUC) values reflecting the timing and progression of seizure activity. The PTZ-treated group showed significantly higher seizure scores than the control group (*p* < 0.05). Likewise, AUC values were also notably higher in the PTZ group, indicating greater seizure severity and longer duration (*p* < 0.05). Conversely, the CAPE+PTZ, 10-HDA+PTZ, and CAPE+10-HDA+PTZ groups showed significantly lower seizure scores than the PTZ group (*p* < 0.05). AUC values were significantly lower in the PC, CAPE+PTZ, 10-HDA+PTZ, and CAPE+10-HDA+PTZ groups than in the PTZ group (*p* < 0.05). No statistically significant differences in AUC were detected among these four groups (*p* > 0.05), although their values remained significantly higher than those of the control group (*p* < 0.05).

#### 3.2.2. Novel Tank Diving Test

**Figure 2:**
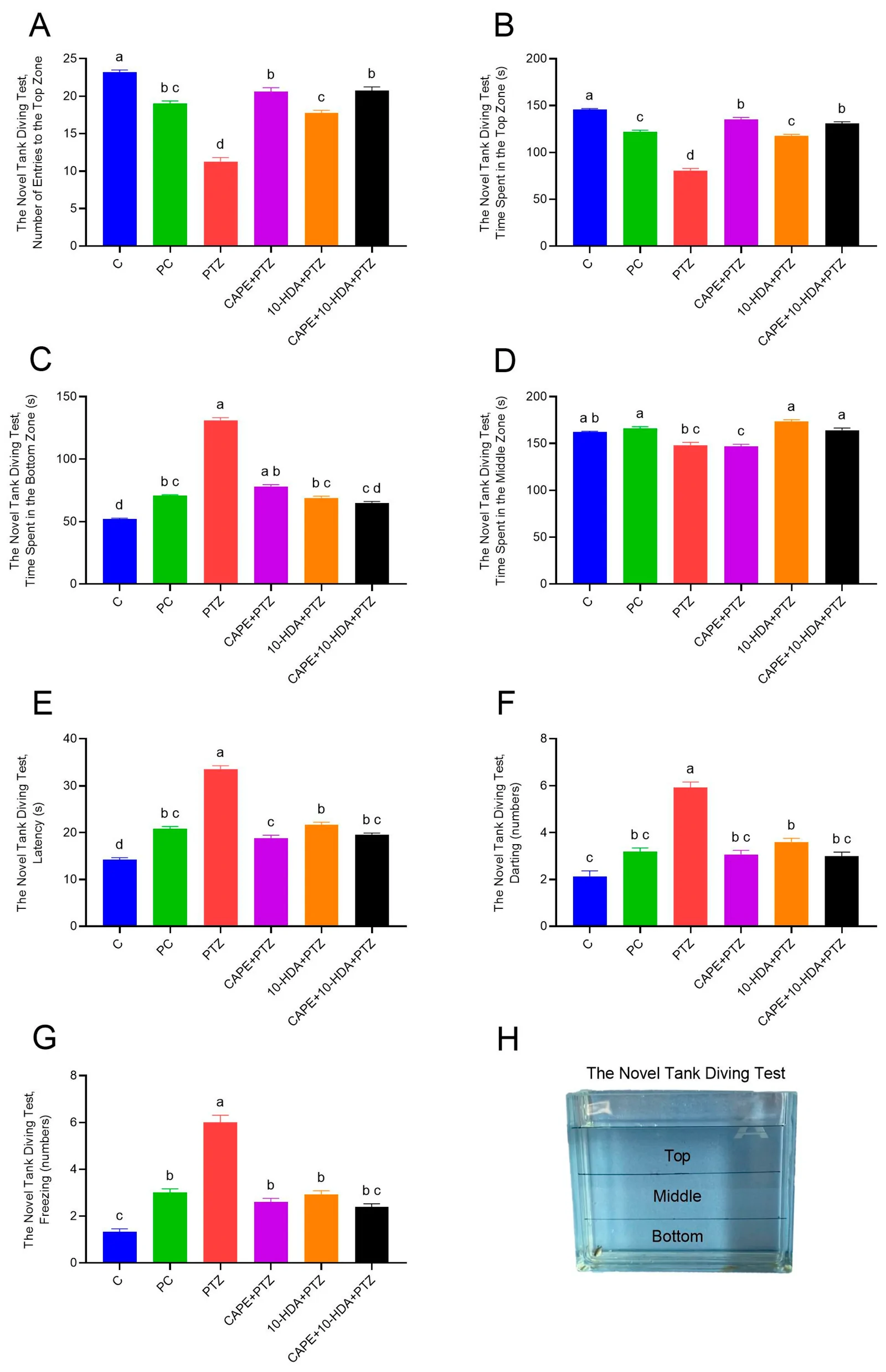
Novel tank diving test results in the control and PTZ-challenged groups. (**A**) Number of entries into the top zone; (**B**) time spent in the top zone (s); (**C**) time spent in the bottom zone (s); (**D**) time spent in the middle zone (s); (**E**) latency to first entry into the top zone (s); (**F**) number of darting events; (**G**) number of freezing episodes; and (**H**) experimental setup of the novel tank test. Time measurements are expressed in seconds, and entries, darting events, and freezing episodes are reported as counts. Bars represent means, and error bars indicate SEM. All 90 fish per group were assessed (30 fish in each of three experimental replicates). Within each of panels (**A**–**G**), groups sharing at least one lowercase letter do not differ significantly, whereas groups with no letters in common differ significantly (*p* < 0.05).

Figure 2 shows the results of the novel tank diving test. In the PTZ group, entries into the top zone and time spent there were significantly lower than in the control group (*p* < 0.05), while bottom-dwelling time, latency, darting, and freezing were significantly higher (*p* < 0.05). These findings suggest increased anxiety-like and seizure-related behaviors after PTZ exposure. The CAPE+PTZ, 10-HDA+PTZ, and CAPE+10-HDA+PTZ groups showed more top-zone entries, longer time in the top zone, and shorter latency to first top-zone entry than the PTZ group (*p* < 0.05). Bottom-dwelling time was significantly lower in the PC, 10-HDA+PTZ, and CAPE+10-HDA+PTZ groups than in the PTZ group, whereas the difference between CAPE+PTZ and PTZ was not statistically significant. For top-zone entries, no statistically significant differences were detected between the PC group and the CAPE+PTZ, 10-HDA+PTZ, or CAPE+10-HDA+PTZ groups (*p* > 0.05). Darting and freezing were significantly reduced in the PC, CAPE+PTZ, 10-HDA+PTZ, and CAPE+10-HDA+PTZ groups compared with the PTZ group (*p* < 0.05). For these two endpoints, no statistically significant differences were detected between the PC group and the supplemented PTZ-challenged groups (*p* > 0.05). Overall, the CAPE+PTZ, 10-HDA+PTZ, and CAPE+10-HDA+PTZ groups showed improvements in anxiety-related behavioral measures compared with the PTZ group.

#### 3.2.3. Y-Maze Test

**Figure 3:**
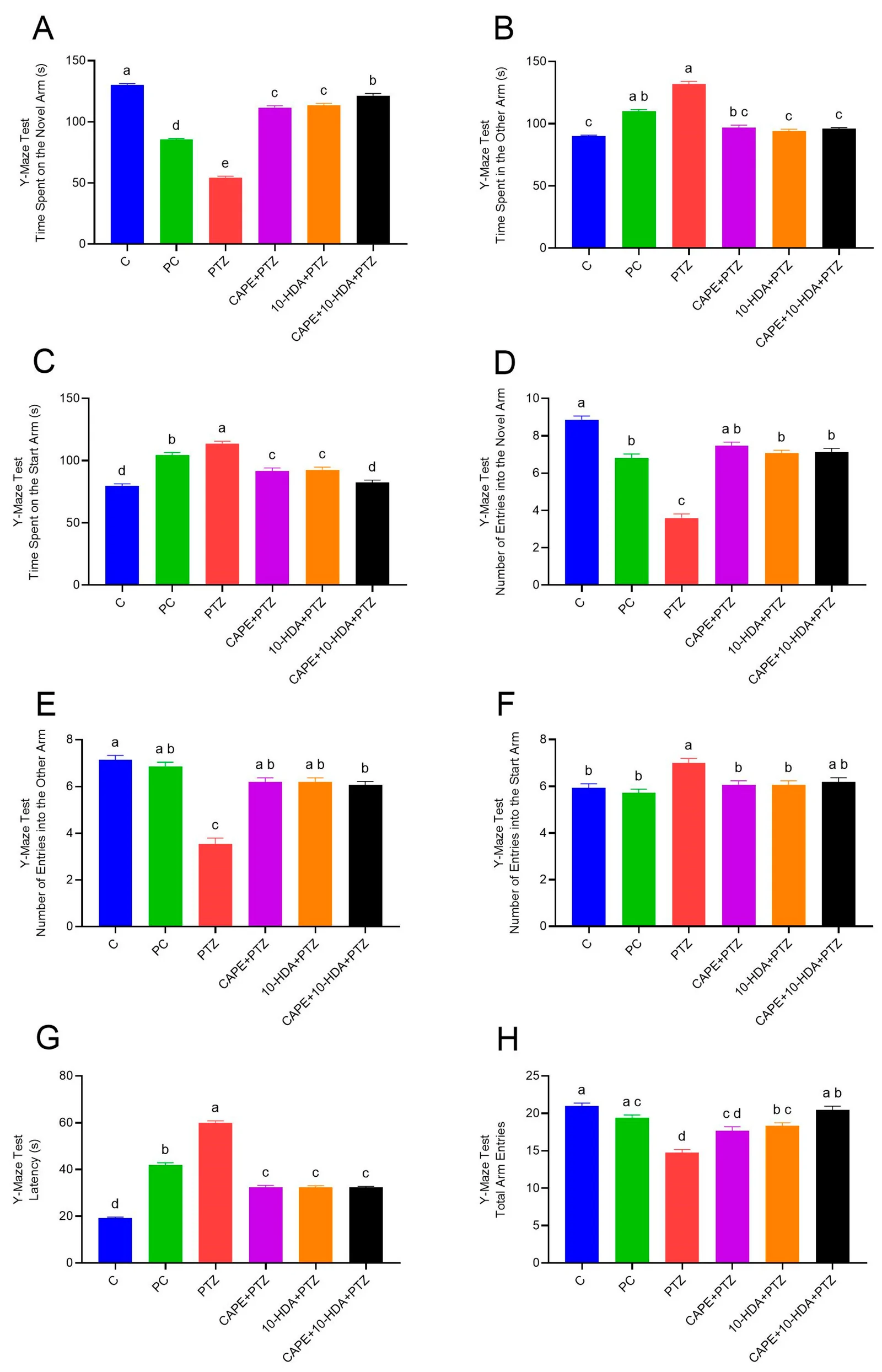
Y-maze test results in the control and PTZ-challenged groups. (**A**) Time spent in the novel arm (s); (**B**) time spent in the other arm (s); (**C**) time spent in the start arm (s); (**D**) number of entries into the novel arm; (**E**) number of entries into the other arm; (**F**) number of entries into the start arm; (**G**) latency to first entry into the novel arm (s); and (**H**) total number of arm transitions. Time measurements are expressed in seconds, and entry and transition measurements are counts. Bars represent means, and error bars indicate the standard error of the mean (SEM). All 90 fish per group were assessed (30 fish in each of three experimental replicates). Within each panel, groups sharing at least one lowercase letter do not differ significantly, whereas groups with no letters in common differ significantly (*p* < 0.05).

Figure 3 presents the Y-maze results. Compared with the control group, the PTZ group spent less time in the novel arm, made fewer novel-arm entries, spent more time in the start arm, and showed a longer latency to first novel-arm entry (*p* < 0.05). Total arm transitions were also lower in the PTZ group (*p* < 0.05).

Compared with the PTZ group, all three supplemented PTZ-challenged groups spent more time in the novel arm, spent less time in the start arm, made more novel-arm entries, and showed shorter latency (*p* < 0.05). Total arm transitions were significantly higher in the 10-HDA+PTZ and CAPE+10-HDA+PTZ groups than in the PTZ group, whereas the difference between CAPE+PTZ and PTZ was not statistically significant.

The combined group spent significantly more time in the novel arm and less time in the start arm than either single-compound group (*p* < 0.05). However, novel-arm entry counts and latency did not differ significantly among the three supplemented groups (*p* > 0.05). Total arm transitions were higher in the combined group than in CAPE+PTZ (*p* < 0.05), but did not differ significantly from 10-HDA+PTZ. Compared with PC, all three supplemented groups spent more time in the novel arm, spent less time in the start arm, and showed shorter latency (*p* < 0.05), while novel-arm entries did not differ significantly. For total arm transitions, only the combined group differed significantly from CAPE+PTZ; none of the three supplemented groups differed significantly from PC.

Time spent in the other arm was lower in all three supplemented groups than in the PTZ group (*p* < 0.05), while entries into that arm were higher (*p* < 0.05). Start-arm entries were lower in the CAPE+PTZ and 10-HDA+PTZ groups than in the PTZ group (*p* < 0.05), but the combined group did not differ significantly from PTZ for this measure.

## 4. Discussion

Adult zebrafish fed CAPE, 10-HDA, or their combination showed lower behavioral seizure scores and better performance in the novel tank and Y-maze tests after PTZ exposure. Assessing these outcomes together let us examine seizure-like behavior and subsequent exploration in the same experiment. The CAPE findings build on earlier evidence from experimental seizure models, while the results for 10-HDA add to the limited knowledge of its neurobehavioral effects. The combined treatment also allowed us to examine whether the responses differed from those observed with either compound alone. These findings warrant further investigation of how changes in seizure severity and exploratory behavior are related.

The pronounced increase in tonic–clonic seizure scores following PTZ exposure confirmed the successful induction of a robust seizure-like phenotype in adult zebrafish. The PTZ model is widely used in zebrafish epilepsy research because it produces a reproducible progression of motor abnormalities that can be quantified behaviorally and pharmacologically modulated by established antiseizure agents [27,28,31,58]. In adult zebrafish, PTZ-induced behavioral manifestations typically progress from increased and disorganized locomotor activity to circular swimming, loss of posture, clonic contractions, and more severe tonic seizure-like states [31]. The temporal progression toward severe motor manifestations observed in the present study closely followed this characteristic pattern. Moreover, the sensitivity of PTZ-induced behavioral responses to established antiseizure drugs supports the use of these endpoints for pharmacological screening [58,59,60]. Recent developments in automated behavioral analysis and machine-learning-based seizure detection have further reinforced the value of zebrafish motor phenotypes as quantifiable measures of seizure activity [61,62,63,64].

Against this well-defined PTZ-induced phenotype, dietary CAPE and 10-HDA markedly attenuated seizure activity. The CAPE+PTZ, 10-HDA+PTZ, and CAPE+10-HDA+PTZ groups exhibited lower tonic–clonic seizure scores than the PTZ group across the observation period, indicating reduced progression toward severe seizure manifestations. This effect was further supported by the AUC analysis. Whereas seizure scores represent behavioral severity at discrete time points, integration of the seizure score–time profile captures the cumulative burden of seizure activity throughout the observation period. Accordingly, the markedly elevated AUC in the PTZ group reflected the magnitude and persistence of the induced seizure phenotype, whereas the lower AUC values following CAPE and/or 10-HDA supplementation indicated an overall reduction in seizure burden rather than an effect confined to a single observation point. The parallel changes in seizure scores and AUC therefore provide complementary evidence that both compounds mitigated the behavioral expression of PTZ-induced seizures. This interpretation is consistent with the established pharmacological responsiveness of the zebrafish PTZ model and with recent approaches emphasizing temporal characterization of seizure-associated behavior rather than reliance on isolated motor observations alone [58,62,64].

The protective effect observed for CAPE is supported by previous evidence from mammalian seizure models. Ilhan et al. [40], demonstrated that CAPE ameliorated seizure severity in PTZ-challenged mice, providing direct evidence for its protective activity against chemically induced seizures. CAPE has also been reported to reduce neuronal injury following PTZ-induced status epilepticus and, more recently, to protect against nicotine-induced seizures [41,42]. Thus, these findings extend the seizure-related effects of CAPE to an adult zebrafish model and show that dietary supplementation attenuates both the instantaneous severity and cumulative behavioral burden of PTZ-induced seizures. In contrast, direct evidence for seizure-modulating effects of 10-HDA remains scarce, making the reduction in seizure scores and AUC observed in the 10-HDA+PTZ group particularly noteworthy. Although the CAPE+10-HDA+PTZ group also showed substantial attenuation of seizure activity, the present experimental design does not permit interpreting this response as pharmacological synergy. Rather, the findings demonstrate that co-administration retained a protective behavioral effect against PTZ challenge. Collectively, these results support the seizure-attenuating potential of CAPE and provide new evidence that 10-HDA may also modulate the behavioral expression of PTZ-induced seizures, warranting further investigation of 10-HDA in dedicated experimental seizure models.

The novel tank diving test revealed that the behavioral consequences of PTZ exposure extended beyond overt seizure activity to a broader anxiety-like phenotype. This assay is widely used in adult zebrafish to assess responses to novelty and acute stress, with vertical exploration, bottom-dwelling, latency to enter the upper zone, freezing, and darting commonly interpreted as informative behavioral endpoints of anxiety-related states [65,66,67,68]. In the present study, PTZ exposure markedly reduced upper-zone exploration while increasing bottom-dwelling time, latency to first upper-zone entry, darting, and freezing. This pattern is consistent with a shift toward a more defensive and stress-associated behavioral state. Similar approaches have demonstrated that reduced vertical exploration and prolonged bottom preference are sensitive to anxiolytic manipulation and environmental stressors in zebrafish, although interpretation should consider the broader locomotor context and the inherent variability of individual behavioral responses [32,33,34,35,69].

Against this PTZ-induced behavioral disturbance, CAPE and 10-HDA supplementation improved several novel tank endpoints. The CAPE+PTZ, 10-HDA+PTZ, and CAPE+10-HDA+PTZ groups showed greater upper-zone exploration, shorter latency, and reduced bottom-oriented behavior relative to the PTZ group, while darting and freezing were also attenuated. These changes indicate differences in exploration and movement during the novel tank test, alongside the lower seizure scores observed in the supplemented groups. These endpoints are sensitive to the established sensitivity of the novel tank test to pharmacological modulation of anxiety-like behavior, as shown for classical anxiolytics such as buspirone, chlordiazepoxide, and diazepam, as well as for other neuroactive compounds tested in adult zebrafish [66,70,71,72].

Fish receiving CAPE or 10-HDA, alone or in combination, showed lower seizure scores, greater upper-zone exploration, and less freezing and darting than fish exposed to PTZ alone. These findings suggest that reduced seizure severity may have helped fish swim and explore more readily after PTZ exposure. The behavioral pattern is also consistent with reduced anxiety-like responses. However, the novel tank test cannot clearly distinguish changes in anxiety from changes in swimming ability, particularly soon after a seizure challenge. The results therefore suggest a possible relationship between lower seizure severity and improved exploration, but further studies are needed to determine whether the compounds also act directly on anxiety.

The present findings also fit with a growing recognition that the novel tank test captures a complex behavioral phenotype influenced by stress reactivity, exploratory drive, housing conditions, sex, and individual variability [33,34,73,74,75]. Recent work has further emphasized that acute stressors can produce distinct and sometimes atypical behavioral responses in this assay, reinforcing the importance of interpreting multiple endpoints together rather than relying on a single variable [76]. In the present study, several novel tank measures differed between the supplemented PTZ-challenged groups and the PTZ group. However, because the supplemented groups without PTZ exposure were not behaviorally assessed, we cannot distinguish attenuation of PTZ-associated changes from independent effects of CAPE and 10-HDA on behavior. Because the novel tank and Y-maze tests were performed immediately after the 60-min seizure observation period, ongoing PTZ effects and post-seizure changes in motor function may have influenced performance. PTZ clearance was not measured, and this interval was not established as a washout period. These findings therefore describe behavioral responses in the acute post-exposure setting and should not be interpreted as evidence of persistent epilepsy-related emotional or cognitive comorbidities.

The Y-maze findings demonstrated that PTZ-induced seizures were accompanied by marked disturbances in cognitive-related behavior in adult zebrafish. Compared with the control group, PTZ-exposed fish spent less time in the novel arm, entered the novel arm less frequently, showed a longer latency to first novel-arm entry, and exhibited fewer overall arm transitions. This pattern is consistent with altered novelty-related exploration and may indicate impaired memory performance, although reduced locomotor activity could also contribute. The Y-maze is an established behavioral paradigm for assessing spatial learning, working memory, and executive-related functions across species, including zebrafish, and its validity in fish is supported by studies demonstrating sensitivity to manipulations of glutamatergic and cholinergic signaling as well as to seizure-associated cognitive dysfunction [56,77,78]. In zebrafish seizure models, PTZ exposure has repeatedly been associated with impairments in learning and memory, supporting the concept that seizure activity can produce cognitive consequences beyond overt motor manifestations [36,79,80]. Related studies have also shown that experimentally induced seizures can disrupt learning processes in both acute and longer-term settings, further reinforcing the vulnerability of cognitive function to seizure activity [81,82].

Fish in the CAPE+PTZ, 10-HDA+PTZ, and CAPE+10-HDA+PTZ groups spent more time in the novel arm, entered it more frequently, reached it sooner, and made more arm transitions than fish in the PTZ group. These results show improved Y-maze performance alongside lower behavioral seizure scores. Previous zebrafish studies have also reported improvements in cognitive-related test outcomes accompanying reductions in seizure activity [79,80]. However, the present results do not separate a specific effect on memory from changes in seizure severity, locomotion, or exploratory activity.

The Y-maze findings can also be considered in the context of the broader experimental epilepsy literature. Mammalian seizure models have shown deficits in spatial learning and memory-related tasks [83,84]. These studies provide context for examining cognitive-related outcomes in zebrafish, but they do not establish the mechanism underlying the differences observed here.

## 5. Conclusions

Dietary CAPE and 10-HDA, administered individually or in combination for 8 weeks, were associated with lower behavioral seizure scores and AUC values in adult zebrafish exposed to PTZ. Supplemented fish also showed greater upper-zone exploration in the novel tank and greater novel-arm exploration in the Y-maze than fish exposed to PTZ alone. The combined group spent more time in the novel arm and less time in the start arm than either single-compound group, although its advantages were not consistent across all endpoints and did not establish pharmacological synergy. These findings support further investigation of both compounds, particularly the less-studied 10-HDA, in experimental seizure models. Further work is needed to distinguish specific anxiety- or memory-related effects from changes in seizure severity, motor function, and baseline behavior.

### Limitations

Interpret the findings in the VPA-treated group with caution. The reported dietary dose represents the nominal amount added to the feed; actual intake and absorption by individual fish were not determined. The study did not measure VPA stability during feed preparation and storage, possible leaching into the water, or blood concentrations. We therefore cannot confirm whether this feeding regimen achieved a therapeutically effective blood concentration. In addition, no VPA dose–response assessment was performed, so an effective dietary dose range was not established for the adult zebrafish PTZ protocol used here.

These limitations restrict the use of the PC group as a benchmark for comparing efficacy. The absence of statistically significant differences between the PC group and supplemented groups for some behavioral measures does not establish equivalent efficacy, and the study was not designed to test equivalence or non-inferiority. Our conclusions about the effects of CAPE and 10-HDA therefore rely primarily on improvements observed relative to the PTZ group, rather than on comparisons with VPA.

## Figures and Tables

**Table 1:** Chemical composition of the diets used in the study.

Groups	%						DE
	Moisture	CP	CF	CrF	CA	NFE	
C	7.55	41.82	8.16	2.43	7.88	32.16	3500
PC	7.48	41.75	8.22	2.51	7.60	32.44	3500
CAPE	7.60	42.00	8.11	2.55	7.67	32.17	3500
10-HDA	7.61	41.88	8.13	2.48	7.70	32.20	3500
CAPE+10-HDA	7.58	41.90	8.23	2.40	7.64	32.25	3500
PTZ	7.52	41.86	8.14	2.47	7.90	32.11	3500
CAPE+PTZ	7.62	41.98	8.13	2.51	7.62	32.14	3500
10-HDA+PTZ	7.63	41.90	8.10	2.46	7.74	32.17	3500
CAPE+10-HDA+PTZ	7.56	41.88	8.21	2.39	7.68	32.28	3500

CP: crude protein; CF: crude fat; CrF: crude fiber; CA: crude ash; NFE: nitrogen-free extract; DE: digestible energy (kcal/kg); (C): control; (PC): 100 µmol/kg feed VPA and 15 mM PTZ; (CAPE): 10 mg/kg feed CAPE; (10-HDA): 10 mg/kg feed 10-HDA; (CAPE+10-HDA): 10 mg/kg feed CAPE and 10 mg/kg feed 10-HDA; (PTZ): control diet and 15 mM PTZ; (CAPE+PTZ): 10 mg/kg feed CAPE and 15 mM PTZ; (10-HDA+PTZ): 10 mg/kg feed 10-HDA and 15 mM PTZ; (CAPE+10-HDA+PTZ): 10 mg/kg feed CAPE with 10 mg/kg feed 10-HDA and 15 mM PTZ.

## Data Availability

The original contributions presented in this study are included in the article/Supplementary Material. Further inquiries can be directed to the corresponding author.

## Supplementary Materials

Video S1: Representative PTZ-induced seizure behaviors in zebrafish used for phenotypic scoring. The video demonstrates the progression of seizure severity from normal swimming activity to severe tonic–clonic manifestations following PTZ exposure (https://www.youtube.com/watch?v=6KnKUwULdSc).; Video S2: Representative recordings of the novel tank diving test in zebrafish, showing the experimental setup and behavioral assessment (https://www.youtube.com/watch?v=6JB0Jn9qOws); Video S3: Representative recording of the Y-maze task in zebrafish demonstrating the experimental setup and assessment of spatial learning and novelty preference (https://www.youtube.com/watch?v=Iq6Ru7WQjBs), accessed on 23 June 2025.
